# Investigation of structural proteins in sea cucumber (*Apostichopus japonicus*) body wall

**DOI:** 10.1038/s41598-020-75580-x

**Published:** 2020-10-30

**Authors:** Yanchao Wang, Mo Tian, Yaoguang Chang, Changhu Xue, Zhaojie Li

**Affiliations:** 1 College of Food Science and Engineering, Ocean University of China, 5 Yushan Road, Qingdao, 266003 China; 2 Laboratory for Marine Drugs and Bioproducts, Qingdao National Laboratory for Marine Science and Technology, Qingdao, 266237 China

**Keywords:** Biochemistry, Biological techniques

## Abstract

Structural proteins play critical roles in the food quality, especially texture properties, of sea cucumbers and their products. Most of the previous studies on sea cucumbers focused on few individual proteins, which limited our understanding of how structural proteins influenced the quality of sea cucumbers. Inspired by the clarification of sea cucumber (*Apostichopus japonicus*) genome, we established an integrated data of structural proteins in the sea cucumber body wall. A portfolio of 2018 structural proteins was screened out from the sea cucumber annotated proteome by bioinformatics analysis. The portfolio was divided into three divisions, including extracellular matrix proteins, muscle proteins, and proteases, and further classified into 18 categories. The presence of 472 proteins in the sea cucumber body wall was confirmed by using a proteomics approach. Moreover, comparative proteomics analysis revealed the spatial distribution heterogeneity of structural proteins in the sea cucumber body wall at a molecular scale. This study suggested that future researches on sea cucumbers could be performed from an integrated perspective, which would reshape the component map of sea cucumber and provide novel insights into the understanding of how the food quality of sea cucumber was determined on a molecular level.

## Introduction

Sea cucumbers, belonging to echinoderm, are consumed as important commercial aquatic products in some Asian countries. As an example, the total production and estimated industrial value of sea cucumbers in China was about 219 kilotons and 7 billion dollars in 2017 respectively^1^. Sea cucumbers have been considered as nutritional foods, and demonstrated various biological activities such as gastric protection, anti-inflammation, anti-diabetes, and moisture-retention activities^2–5^. Due to their biological activities and unique texture properties, sea cucumbers have attracted increasing research interest worldwide.

One important desirability of sea cucumbers comes from their unique texture, which is soft and appropriately elastic. A few studies have reported the texture changes of sea cucumbers during processing, such as boiling and resorption processes^6,7^. Moreover, sea cucumbers are prone to going autolysis and subsequent texture deterioration, which were considered to be caused by some endogenous enzymes, such as cysteine proteinases^8^ and metalloproteinases^9^. Therefore, understanding the molecular mechanism of sea cucumber texture formation could significantly contribute to the quality control of sea cucumbers. Besides, previous studies have demonstrated that sea cucumber body wall is mainly comprised of mutable collagenous tissue, which is a remarkable example of biological materials, being able to rapidly change its stiffness and extensibility under neural control^10,11^.

Structural proteins, being the most abundant component, play critical roles in maintaining the texture properties of sea cucumbers. Sea cucumber body wall, the major edible portion of sea cucumber, is comprised of 40–60% protein (dry weight basis) and acknowledged as protein-rich food material^12^. Collagens account for about 70% of the total protein mass of the body wall and are widely considered as the most important structural proteins of sea cucumbers^13^. Besides, a few other proteins in the sea cucumber body wall have been respectively characterized, including myosin^14^, paramyosin^14^, actin^14^, fibropellin^15^, tenascin^15^, tensilin^16,17^, stiparin^18^, and fibrillin^19,20^.

Foodomics has emerged as a new approach and could be conceptualized as a global discipline to study the food domain as a whole with the combination of omics (proteomics, lipidomics, glycomics, metabonomics, etc.) analytical techniques and bioinformatics^21^. Especially, proteomics studies encompass the qualitative and quantitative characterization of entire protein components in an organism, which could be used to investigate protein-rich food products from an integrated perspective. A considerable number of studies have been conducted on sea cucumbers, however, focused on individual preselected molecules. Recently, the genome of sea cucumber (*Apostichopus japonicus*) have been published in the public database (National Center of Biotechnology Information, NCBI)^22^, which enables one to investigate sea cucumbers by using the omics strategy in a post-genome era. Here, this study aimed to investigate the structural protein components of sea cucumber body wall from an integrated perspective by using a combination of bioinformatics and proteomics approaches, including (1) defining the portfolio of structural proteins from the large dataset of sea cucumber annotated proteome by using bioinformatics tools; (2) confirming the presence of prescreened proteins in the portfolio by using the proteomics-based approach. Furthermore, the spatial distribution heterogeneity of structural proteins in different locations of sea cucumber body wall was investigated by using the comparative proteomics-based approach.

## Results and discussion

### In silico definition of a portfolio of structural proteins in *A. japonicus*

Previous histological studies have demonstrated that sea cucumber body wall is mainly comprised of connective tissue and muscle tissue^8^. While the phenomenon of autolysis has been widely concerned due to its damage to the structure and quality of sea cucumber, which majorly results from the endogenous proteases^9^. Therefore, we suggested classifying the structural proteins of the sea cucumber into three divisions, including extracellular matrix (ECM) proteins, muscle proteins, and proteases (Table 1). Protein components in each division were classified into different categories based on the domain criteria and NCBI annotation. A portfolio of 2018 structural proteins was screened out from *A. japonicus* annotated proteome through in silico analysis, including 1254 ECM proteins, 253 muscle proteins, and 511 proteases. ECM proteins, being important food components, could construct a well-organized three-dimensional structural network. The major constituents of ECM proteins are fibrous-forming proteins, including collagens, proteoglycans, and glycoproteins^23^. Collagens have been verified as the most abundant ECM proteins in the sea cucumber body wall, which play critical roles in the food quality, especially the texture properties, of sea cucumbers and their processing products. Proteoglycans, consisting of a core protein and one or more glycosaminoglycan chains, could interact with collagens and ECM molecules via their core proteins or through their glycosaminoglycan chains and contribute to the formation of ECM scaffolds^23^. Researches have demonstrated that the structure and composition of proteoglycans could strongly influence the mechanical properties and textures of meat products^24^. ECM glycoproteins are associated with collagens and proteoglycans to construct the complex fibrillar structure^23^. Muscles are characterized by individual muscle fibers and the mosaic composition, which contribute to the structural properties of tissues and subsequent food texture^25^. Endogenous proteases could result in the degradation of structural proteins and lead to food texture changes.

**Table 1:** The division, catergory and count of in silico predicted structural proteins of *A. japonicus*.

Division	Category	Protein count in *A. japonicus* annotated proteome
ECM proteins		1254
	Collagens	37
	Proteoglycans	9
	ECM glycoproteins	1208
Muscle proteins		253
	Myosins	55
	Actins	29
	Troponins	3
	Tropomyosin	1
	Paramyosin	1
	Actin-binding proteins	104
	Myosin-binding proteins	15
	Titins	35
	Obscurins	4
	Twitchins	6
Proteases		511
	Aspartic peptidases	7
	Cysteine peptidases	139
	Metallopeptidases	229
	Serine peptidases	127
	Threonine peptidases	9

The diverse bilaterian organisms share a common set of ECM proteins^26^. Naba et al.^27^ established a list of 55 InterPro domains commonly found in ECM proteins of vertebrates. In the present study, ECM proteins in sea cucumber annotated proteome were predicted according to the domain criteria and further classified into three categories, i.e., collagens, proteoglycans, and ECM glycoproteins (Table 1 and Supplementary Data S1). Thirty-four of ECM proteins contain at least one triple helix structure (InterPro domain: IPR008160), which is generally regarded as a collagenous domain. Nineteen of ECM proteins containing triple helix structures are annotated as collagens in NCBI. Fifteen of ECM proteins containing triple helix domains are annotated as glycine-rich protein (PIK60693.1), EMI domain-containing protein (PIK35653.1) and hypothetical proteins respectively. One ECM protein (PIK59149.1) contains a C-terminal domain for fibrillar collagens (IPR000885), which may be the noncollagenous regions of collagens. Two ECM proteins (PIK44932.1 and PIK42190.1), containing VWC domains, are annotated as collagens in NCBI. Previous studies have demonstrated that several collagens contain a VWC module in their N-propeptides^28^. Here, these 37 proteins were categorized as collagens in this current study (Supplementary Data S1).

Based on domain prediction and manual curation, nine of ECM proteins in *A. japonicus* annotated proteome were defined as proteoglycans, including heparan sulfate proteoglycans, agrins, aggrecan, and chondroadherin (Supplementary Data S1). In addition to known collagens and proteoglycans, we categorized 1206 of ECM proteins as ECM glycoproteins, such as laminins, fibronectins, tenascins, thrombospondins, and fibulins, etc. It should be noticed that two tensilin molecules, which were not present in the ECM proteins of vertebrates, were found in sea cucumber annotated proteome. Tensilin has been characterized as a tissue-stiffening protein, contributing to the special mechanical properties of sea cucumber tissues^17^. Hence, a total of 1208 proteins including tensilins were categorized as ECM glycoproteins (Supplementary Data S1). Here, we noted that the assignment of some proteins to ECM glycoproteins may be debated since numerous ECM glycoproteins are annotated as hypothetical proteins in the current proteome database. Since the *A. japonicus* annotated proteome is dynamically updated, the definition of structural proteins might need a revision in the future according to the update of *A. japonicus* genome and proteome databases.

Bioinformatics prediction of muscle proteins was conducted based on the known vertebrate and invertebrate muscle proteins. Vertebrate muscle mainly comprises actomyosin system and auxiliary proteins, including myosins^29^, actins^29^, troponins^29^, tropomyosins^29^, myosin-binding proteins^30^, actin-binding proteins^31^, titins^32^, myozenins^32^, calsarcins^32^, telethonins^32^, myotilins^32^, desmins^32^, myopalladins^32^, obscurins^33^, myomesins^34^, M-proteins^34^, and muscle ankyrin repeat proteins^35^. As shown in Table 1, 246 proteins, belonging to 8 categories were found in sea cucumber predicted proteome. Besides, paramyosin and twitchins, which have been generally identified only in invertebrate muscles, were also found in sea cucumber annotated proteome. Based on the currently available sea cucumber proteome, the above 253 proteins were classified into 10 categories and defined as sea cucumber muscle proteins (Supplementary Data S1).

Proteases represent a large family of enzymes, which may induce the autolysis of sea cucumber structural proteins. A total of 511 proteases, including aspartic peptidases, cysteine peptidases, metallopeptidases, serine peptidases, and threonine peptidases, were screened out from sea cucumber annotated proteome (Table 1 and Supplementary Data S1).

### Structural protein profiling of sea cucumber body wall

**Figure 1:**
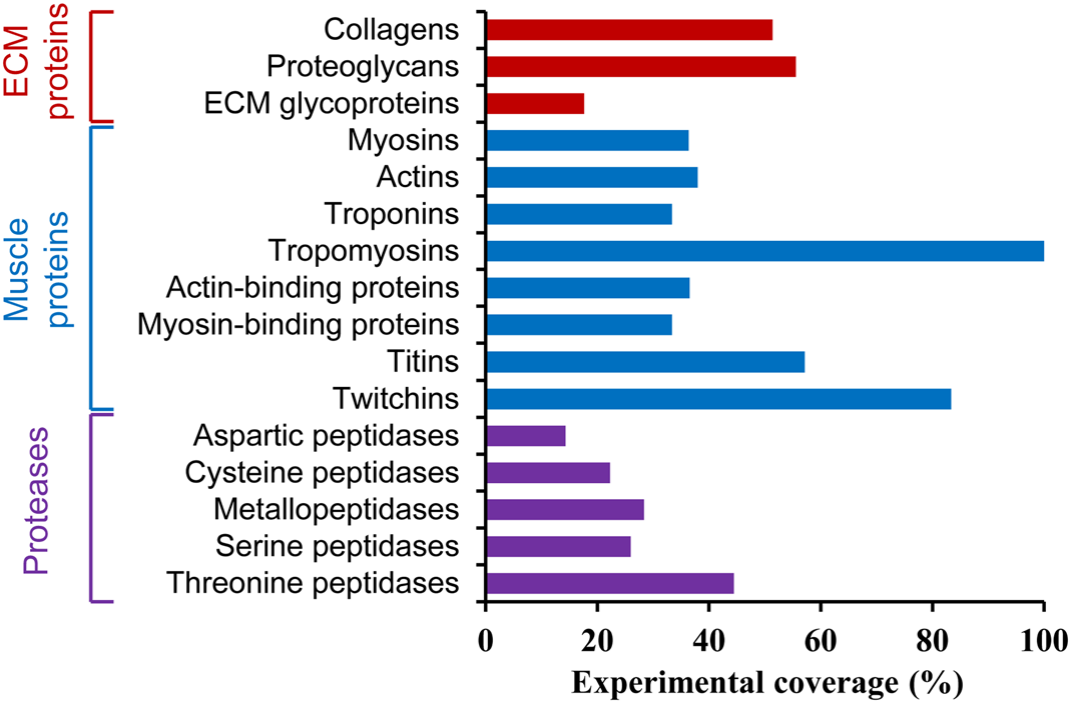
Experimental coverage of in silico identified structural proteins.

The presence of in silico predicted structural proteins in the sea cucumber body wall was confirmed by using an iTRAQ-based proteomics approach. Sixteen of eighteen in silico defined protein categories were detected in the sea cucumber body wall by LC–MS/MS (Fig. 1 and Supplementary Data S2). Compared with in silico predicted structural proteins, the experimental coverages of ECM proteins, muscle proteins, and proteases were 19%, 40%, and 26%, respectively.

**Figure 2:**
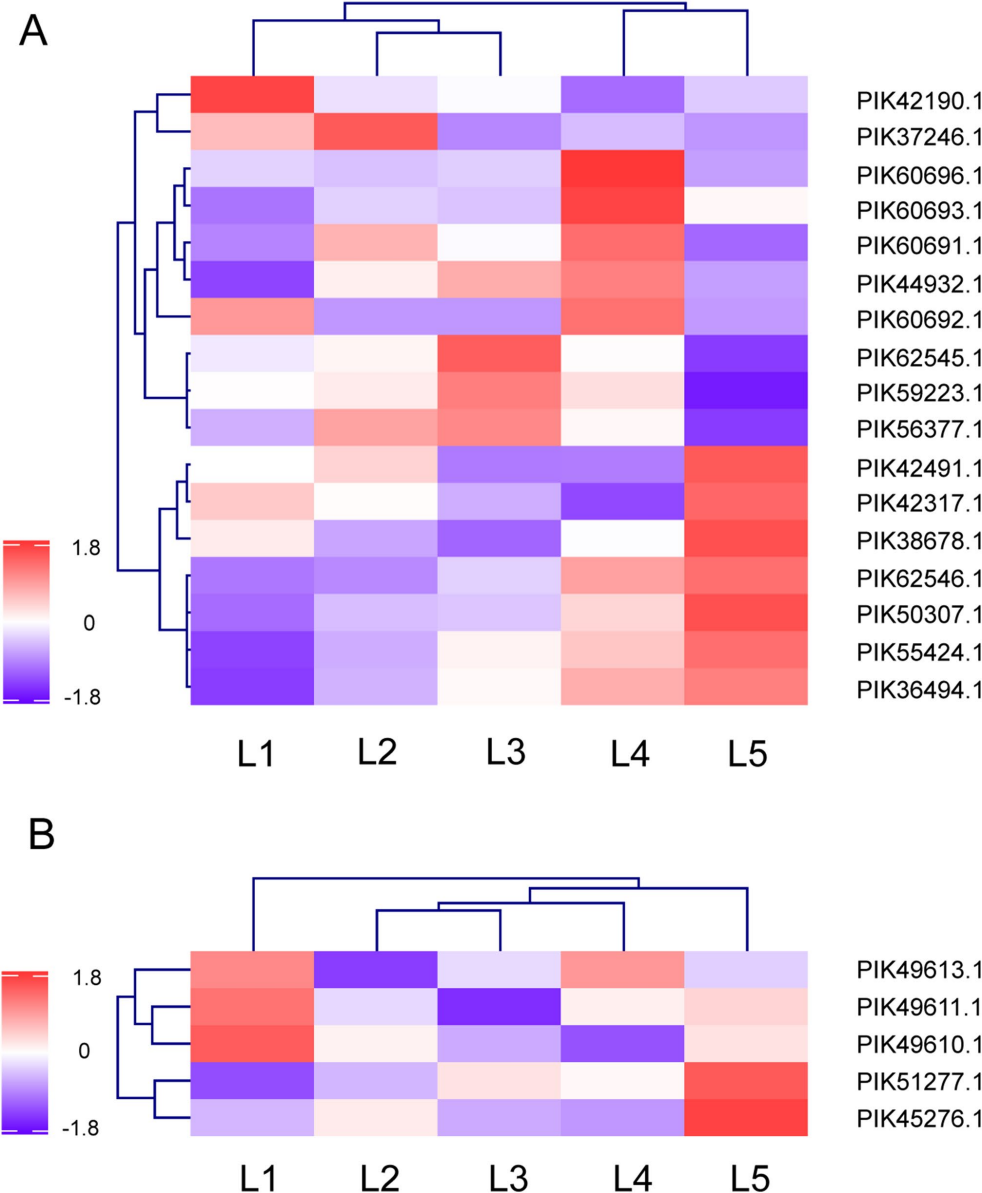
List of collagens and proteoglycans in the sea cucumber body wall detected by using iTRAQ-based mass spectrometry.

Various types of collagens were detected in the sea cucumber body wall, including collagen types I, IV V, IX and XVIII (Fig. 2 and Supplementary Data S2). Collagens could be classified into seven families according to their common domain homology and functions, including fibrillar collagens, network-forming collagens, fibril-associated collagens with interrupted triple helices (FACITs), membrane-associated collagens with interrupted triple helices, anchoring fibrils, beaded-filament-forming collagens, and multiple triple-helix domains and interruptions (MULTIPLEXIN)^23^. Collagen types I and V, which belong to fibrillar collagens, could provide tissues with tensile strength^23^. Collagen type IV which is flexible and capable of interacting with each other, plays an important role in network-forming^23^. Collagen type IX, belonging to FACITs, could interact with fibrillar collagens and link collagen fibers together and with other ECM components^23^. Collagen type XVIII, which possesses a central triple-helical collagenous domain and carries three heparan sulfate chains, could be included into MULTIPLEXIN collagens^36^. Our previous studies have demonstrated that collagen fibrils of sea cucumbers are heterotypic, consisting of various types of collagens^37^. It is well known that different types of collagens are distinct in structure, physical properties, tissue distribution, and functions^38,39^. And most of the previous studies on sea cucumber autolysis and sea cucumber processing (including boiling, drying, soaking, and rehydration) attributed changes in the food properties of sea cucumbers to collagens. The diversity of collagens might play important roles in maintaining the structure and texture of sea cucumbers through interacting with other molecules^37,40^.

Five basement membrane proteoglycans were detected in the sea cucumber body wall (Fig. 2 and Supplementary Data S2). It is well known that basement membrane proteoglycans carry mostly heparan sulfate chains. However, most of the previous studies demonstrated that the major glycosaminoglycan component in the sea cucumber was fucosylated chondroitin sulfate and there has been no report on the presence of heparan in the sea cucumber. Heparan might exist in the sea cucumber body wall with relatively low content and the presence of heparan need to be verified in future researches. Intriguingly, well-known chondroitin sulfate-bearing proteoglycans (aggrecan, versican, neurocan, brevican, biglycan, decorin, betaglycan, syndecan) were not detected in the sea cucumber body wall. Our previous researches revealed that fucosylated chondroitin sulfate is covalently associated with collagen fibrils^40^ and the attachment might be via type IX-like collagens^37^. Results of the proteomics study on proteoglycans were in good agreement with previous conclusions.

**Figure 3:**
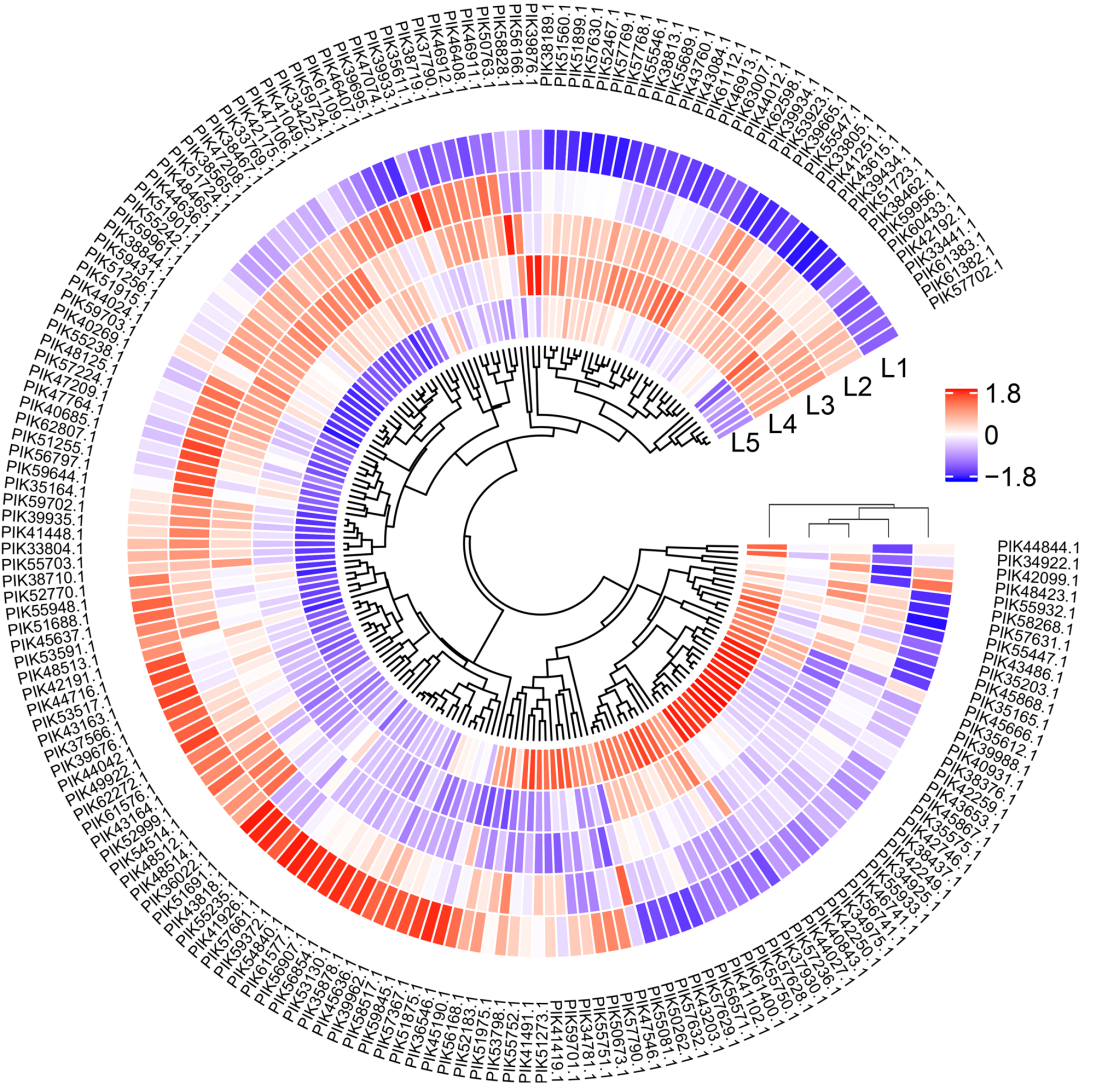
List of major extracellular matrix glycoproteins in the sea cucumber body wall detected by using iTRAQ-based mass spectrometry.

A total of 213 of predicted ECM glycoproteins were detected in the sea cucumber body wall, including fibrillins, laminins, fibronectins, tensilins, fibrinogens, and fibulins (Fig. 3 and Supplementary Data S2). Elastic fibers, consisting of elastin and microfibrils, are acknowledged as large ECM structures in most of the vertebrates and provide elastic recoil to tissues^23^. Nevertheless, due to the absence of elastin, microfibrils and supra-microfibrillar arrangement provide essential elastic recoil to tissues in invertebrates such as sea cucumbers^41^. Fibrillins are the major constituents of microfibrils and most likely perform structural roles in the formation of microfibril scaffold^23^. Besides, some other fibrous-forming glycoproteins, i.e., laminins, fibronectins, fibrinogens, and fibulins, were detected, which have not been previously reported in sea cucumbers. Tensilin has been characterized as a fibril-aggregating and tissue-stiffening protein of sea cucumbers, which was also detected in the current study. The presence of various ECM glycoproteins in the body wall contributed to the formation of sea cucumber mutable collagenous tissue with special mechanical properties.

**Figure 4:**
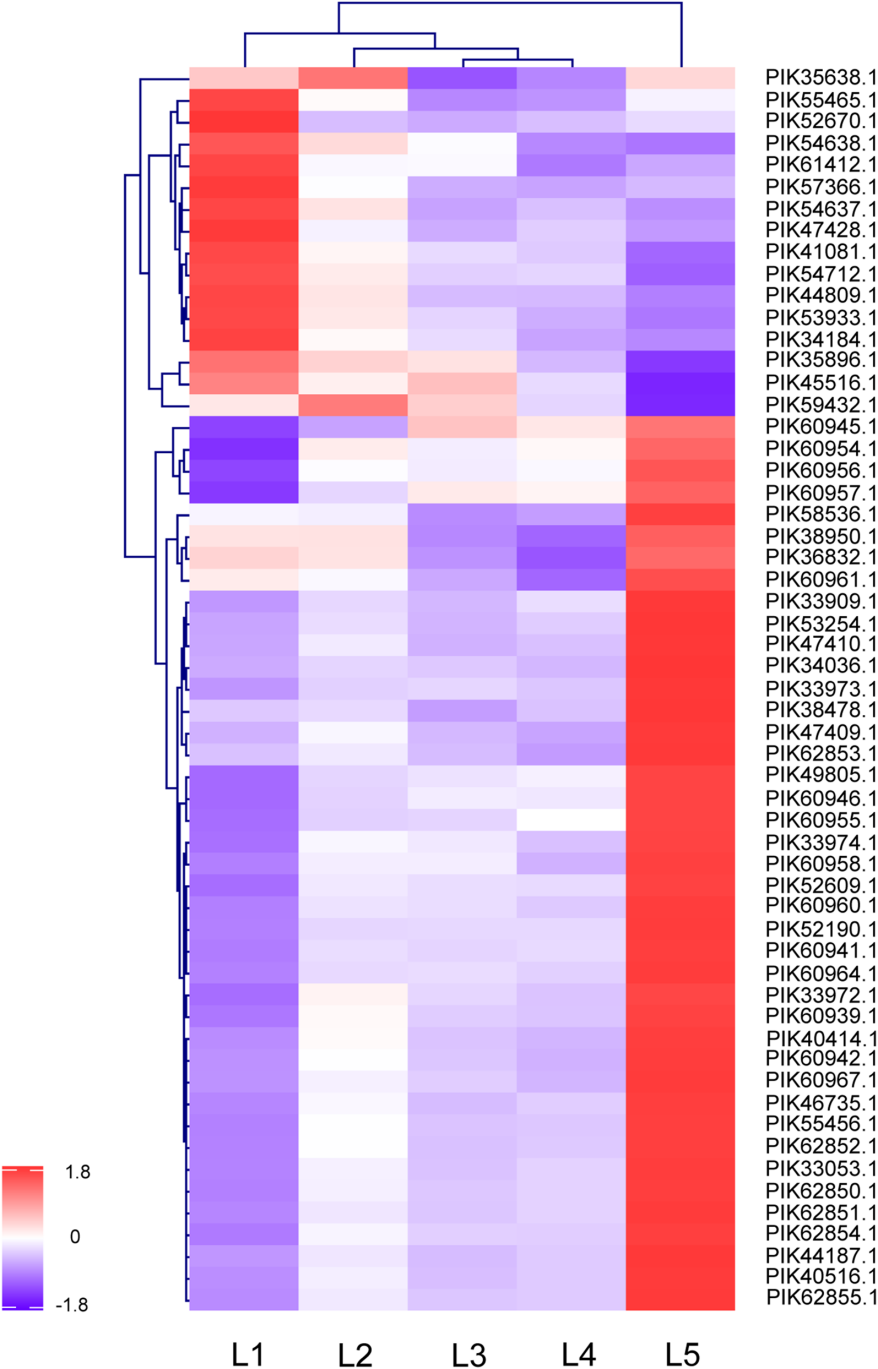
List of muscle proteins with signficantly different abundances (fold change ≥ 1.5 or ≤ 0.67) among samples from different spatial locations of sea cucumber body wall detected by using iTRAQ-based mass spectrometry.

Most of the predicted muscle proteins in the portfolio were detected in the iTRAQ experiments except for paramyosin and obscurin (Fig. 1 and Supplemental Data S2). Previous researches have confirmed the presence of distinct muscles associated with the body wall and scattered myocytes inserted in the body wall by microscope examination^42,43^. Muscle proteins were detected in samples from outer to inner layers of the sea cucumber body wall (Fig. 4). It suggested that, besides ECM fibril networks, muscle protein-forming networks might also involve in the texture formation of sea cucumbers.

Based on LC–MS/MS analysis, far beyond the reported proteases were detected in the sea cucumber body wall, including 33 serine peptidases, 65 metallo peptidases, 31 cysteine peptidases, four threonine peptidases, and one aspartic peptidase (Supplementary Data S2). The presence of various proteases with relatively high abundances, such as aspartic peptidase (PIK49084.1), cysteine peptidases (PIK48449.1, PIK52423.1), metallo peptidases (PIK34919.1) and serine peptidase (PIK42881.1), suggested that endogenous proteases played critical roles in the autolysis of sea cucumbers. Ready-to-eat sea cucumber products, which are prepared by treatment of fresh sea cucumbers with high temperature or high pressure, have become popular due to their convenience. However, ready-to-eat sea cucumber products would undergo texture-softening and subsequent deterioration of quality during their storage. The softening of texture might result from the presence of some thermostable proteases in the sea cucumber body wall besides non-enzymatic degradation.

Each protein component involved in the structure formation might contribute to the unique texture of the sea cucumber body wall. It is becoming evident that structural protein components require partners to generate complex structural networks with other macromolecules including the remodeling of proteases. The integrated data of structural proteins provided an integrated protein component map of sea cucumbers.

### Comparative protein mapping of sea cucumber body wall

The spatial distribution of structural proteins in different locations of sea cucumber body wall was characterized based on the proteomics approach. For ECM proteins, 96 proteins showed significantly different abundances in samples from different spatial locations, including one proteoglycan, six collagens, and 89 glycoproteins (Supplementary Data S3). For muscle proteins, 57 components showed significantly different abundances in samples from differing spatial locations of sea cucumber body wall (Supplementary Data S3). A set of 50 proteases showed significantly different abundances in samples from differing locations, including 12 members from the serine peptidase family, 24 members from the metallo peptidase family, 12 members from the cysteine peptidase family, one from the threonine peptidase family, and one from the aspartic peptidase family (Supplementary Data S3).

Various muscle proteins showed high abundances in samples from the inner layer (L5) (Fig. 4 and Supplementary Data S3), which was consistent with the presence of an inner circular muscle layer in the body wall. Striated-type myosins and striated-type myosin binding proteins were observed, which implied the presence of striated-type muscles in the sea cucumber. Nevertheless, some typical proteins in vertebrate sarcomere were not observed in the sea cucumber body wall. Vertebrate myofibrils comprise an intact troponin system (troponin C/T/I), while troponin I and troponin T were observed in the sea cucumber annotated proteome, and only troponin I was confirmed in the experimental investigation. The absence of troponin C revealed certain difference might exist between sea cucumber muscles and vertebrate striated muscles. Moreover, myosin light-chain kinase proteins and calponins were detected, which were present in smooth muscles. The absence of complete troponins and the presence of myosin light chain kinases and calponins suggested that the contraction regulation of sea cucumber muscles might resemble that of smooth muscles. This suggested that sea cucumber muscles might be hybrid. Hybrid muscles have been observed in other invertebrate muscles, such as sea anemone^29^. Striated muscles and smooth muscles exhibited distinct elastic properties due to their functional and structural difference^44^. The proteomic analysis of muscle proteins revealed that both striated and smooth muscle protein components existed in the sea cucumber body wall, contributing to the texture and mechanical properties of sea cucumbers.

**Figure 5:**
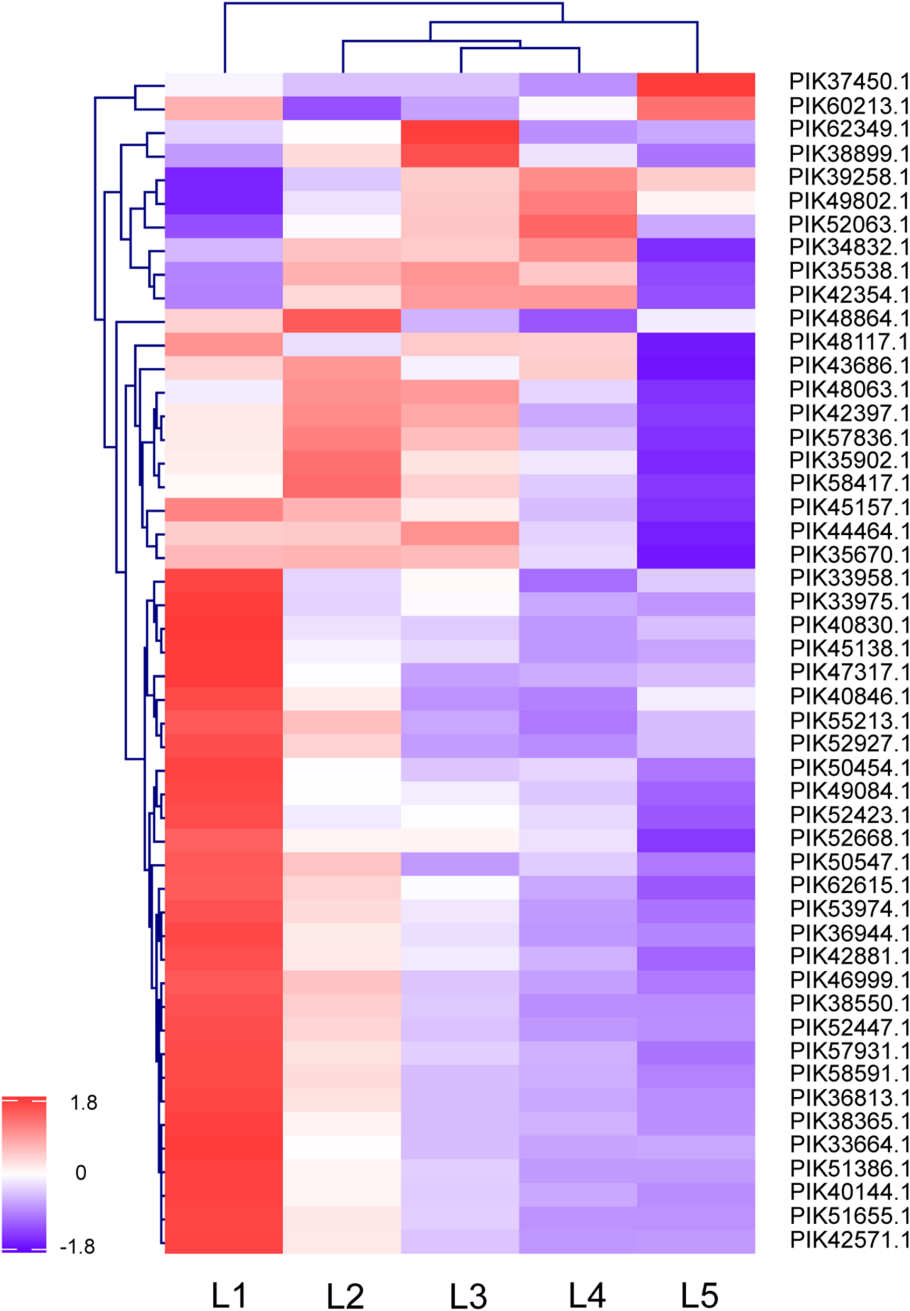
List of proteases with signficantly different abundances (fold change ≥ 1.5 or ≤ 0.67) among samples from different spatial locations of sea cucumber body wall detected by using iTRAQ-based mass spectrometry.

Protein abundances of various proteases significantly increased along with samples from inner to outer layers (i.e., L5 to L1) (Fig. 5 and Supplementary Data S3). It is acknowledged that sea cucumbers immediately undergo autolysis due to the existence of endogenous proteases. Previous studies have observed that structural damage in the outer dermis layer was more pronounced in comparison to the inner layer during autolysis^8^. Here, the results indicated that larger contents of proteases existed in outer layers in comparison to inner layers of sea cucumber body wall. The severe autolysis of sea cucumbers might relate to several types of proteases with considerable abundances and result from a combined action of various proteases. Although activities of identified proteases were not determined in the current study, the comparative profiling of enzymes in the body wall revealed the discrepant presence of various proteases at a whole scale. Hence, the autolysis behavior of sea cucumbers could be investigated from an integrated perspective and different types of proteases should be concerned to control the autolysis of sea cucumbers in the future.

Most of the previous studies on biomacromolecules of sea cucumbers mainly focused on collagens. Here, we demonstrated the presence of various fibrous-forming structural proteins, i.e., collagens, proteoglycans, glycoproteins, and muscle proteins, which suggested that collagen fibrils, fibrillin microfibrils, and myofibrils participated in the complex structure formation of sea cucumber body wall. Besides, various types of proteases with considerable abundances were detected in the sea cucumber body wall, which might play an important role in the structural remodeling and autolysis of sea cucumbers. This study confirmed that there were various structural proteins in the sea cucumber body wall and defined a portfolio of these structural proteins, including both major proteins and minor proteins, which all may contribute to the unique texture and mechanical properties of sea cucumber. By taking various types of structural protein components into consideration, one would obtain a deeper insight into the food quality of sea cucumbers, and it would facilitate the development of novel strategies to control and improve the quality.

## Conclusion

In conclusion, this study proposed a portfolio of structural proteins in the sea cucumber and explored their spatial distribution in the body wall, by using a combination of bioinformatics and proteomics approaches. The portfolio of 2018 in silico predicted structural proteins were classified into three divisions, i.e., ECM proteins, muscle proteins, and proteases. The presence of 472 structural proteins was confirmed, which were included into 16 categories, including collagens, proteoglycans, glycoproteins, myosins, actins, troponins, tropomyosins, actin-binding proteins, myosin-binding proteins, titins, twitchins, aspartic peptidases, cysteine peptidases, metallopeptidases, serine peptidases, and threonine peptidases. A significant distribution discrepancy of muscle proteins and proteases was observed in differing spatial locations of sea cucumber body wall. The portfolio of structural proteins would serve as an infrastructure for researchers working on sea cucumbers, which might result in a better understanding and controlling of the food quality of sea cucumber.

## Material and methods

### Bioinformatics analysis

Structural proteins in the sea cucumber body wall were classified into three divisions, including extracellular matrix (ECM) proteins, muscle proteins, and proteases. Sea cucumber (*A. japonicus*) annotated proteome (ASM275485v1, updated by NCBI in November 2017) was downloaded from the NCBI database. The characteristic domains of proteins were obtained by using the Interproscan software^45^. Core ECM proteins in sea cucumber annotated proteome were predicted according to a previously published domain-based protocol by Naba et al.^27^. The lists of muscle proteins and proteases were defined manually based on previously published literature. Based on the defined protein lists, these two divisions of proteins (muscle proteins and proteases) in sea cucumber annotated proteome were predicted according to the protein annotation and characteristic InterPro domains.

### Sample collection

Fresh sea cucumber (*A. japonicus*) was purchased from a local aquatic market in Qingdao, China. Sea cucumbers were immediately dissected, gutted and cleaned with distilled water at 4 °C The attached viscera and longitudinal muscle layers on the inside were pulled off. Both the pigmented outer dermis and circular muscles were included in the body wall. Samples were taken from the central part of the body wall along the longitudinal axis. The sectioned body wall was divided into five equal portions perpendicular to the longitudinal axis (Supplementary Figure S1). The thickness of the samples was kept constant to 5 mm. Samples from the outer layer to the inner layer were named L1 (the outer layer with pigmented dermis), L2 (the second outer layer), L3 (the intermediate layer), L4 (the second inner layer), and L5 (the inner layer with circular muscles), respectively. All samples were frozen immediately with liquid nitrogen and then stored at − 80 °C before protein extraction.

### Protein extraction and isobaric tag for relative and absolute quantitation (iTRAQ) labeling

Protein extraction was performed according to published protocols with minor modification^46^. The sample was ground to powder following freezing in liquid nitrogen and suspended in the lysis buffer (100 mM NH_4_HCO_3_, 6 M Urea, 0.2% SDS, pH 8.0). The suspension was sonicated for 5 min and centrifugated at 12,000*g* for 15 min at 4 °C The supernatant was transferred to another clean tube. Dithiothreitol was added into the supernatant at a final concentration of 10 mM, and the suspension was incubated at 56 °Cfor 1 h. Then, iodoacetamide was added at a final concentration of 55 mM and incubated for 1 h in the dark to allow alkylation. Subsequently, the suspension was mixed with cold acetone (1/4, v/v), left at − 20 °C for 2 h, and centrifugated at 12,000*g* for 15 min. The precipitation was collected, washed with cold acetone, and resuspended in the dissolution buffer (100 mM triethylammonium bicarbonate, 6 M urea, pH 8.5). The total protein concentration was determined using the Bradford method with bovine serum albumin as a standard. For each sample, a total of 120 µg of protein was digested with trypsin (Promega, Madison, WI, USA) overnight at 37 °C setting a protein/trypsin ratio of 40:1. Following trypsin digestion, the resulting peptides were desalted using a C18 column (Thermo, Waltham, MA, USA) and then freeze-dried.

The iTRAQ labeling method was performed as previously reported with minor modifications^47^. Peptides were dissolved in 1 M triethylammonium bicarbonate solution and labeled by 8-plex iTRAQ reagent (Sigma, St. Louis, MO, USA) following the manufacture’s protocol. Peptides were labeled with iTRAQ reagents for 2 h at room temperature. Peptide labeling was performed on five samples (L1: 115, L2: 116, L3: 117, L4: 118, L5: 119). After iTRAQ labeling, samples were pooled, desalted and freeze-dried.

### Peptide fractionation and liquid chromatography-tandem mass spectrometry (LC–MS/MS) analysis

The fractionation was performed by using a Rigol L-3000 HPLC system (RIGOL, Beijing, China) coupled to a Waters BEH C18 column (4.6 × 250 mm, 5 μm, Waters, Milford, MA, USA). Peptides were dissolved in 1 mL of buffer A (2% acetonitrile, 98% H_2_O, pH 10.0) and separated at a flow rate of 1 mL/min. The temperature was maintained at 50 °C. A binary gradient elution system, comprising buffer A and buffer B (98% acetonitrile, 2% H_2_O, pH 10.0), was applied as follows: 3–5%B from 0 to 10 min, 5–20%B from 10 to 30 min, 20–40%B from 30 to 48 min, 40–50%B from 48 to 50 min, 50–70%B from 50 to 53 min, 70–100%B from 53 to 54 min, 100%B from 54 to 58 min, 100–3%B from 58 to 60 min, 3%B from 60 to 70 min. The absorbance wavelength was 214 nm, and the eluent was collected at 1-min interval. Eluted peptides were pooled into 10 fractions and freeze-dried.

LC–MS/MS analysis was performed by using an EASY-nLC 1200 UHPLC system (Thermo, Waltham, MA, USA) coupled to a Q Exactive HF-X mass spectrometer (Thermo, Waltham, MA, USA) with a Nanospray Flex ESI source. The system was equipped with a C18 trap column and a C18 analytical column (150 µm × 150 mm, 1.9 µm) made in-house. Each fraction was dissolved in buffer C (100% H_2_O, 0.1% formic acid) and separated at a flow rate of 600 nL/min. A binary gradient elution system, comprising buffer C and buffer D (80% acetonitrile, 20% H_2_O, 0.1% formic acid), was applied as follows: 5–10%D from 0 to 2 min, 10–30%D from 2 to 51 min, 30–50%D from 51 to 53 min, 50–90%D from 53 to 55 min, 90–100%D from 55 to 60 min, 100%D from 60 to 64 min, 100–5%D from 64 to 65 min, 5%D from 65 to 75 min.

Data were acquired with the following MS conditions: ESI source, positive mode; acquisition mode, data-dependent top 40; ion spray voltage, 2.3 kV; capillary temperature, 320 °C; MS scan range 407–1500 m/z; MS scan resolution, 60,000 (200 m/z); MS/MS scan resolution, 15,000 (200 m/z); normalized collision energy, 32%; dynamic exclusion, 20 s.

### Data processing and analysis

Raw data files were searched using Proteome Discoverer 2.2 against *A. japonicus* annotated proteome (ASM275485v1) database downloaded from NCBI^22^. The search parameters were set as follows: enzyme specificity was set to trypsin with two missed cleavage sites; the fixed modification was carbamidomethyl (C); N-terminal modifications were iTRAQ 8-plex (N-terminal) and acetyl (N-terminal); potential dynamic modifications were oxidation (M) and iTRAQ 8-plex (K, Y); precursor mass tolerance was set to 10 ppm; fragment mass tolerance was set to 0.02 Da. Peptides with a 99% confidence interval and proteins containing at least one unique peptide were used for protein identification. Only proteins that contained at least two peptides were used for protein quantification. Protein identification results were filtered with a false discovery rate (FDR) ≤ 1% and quantification results were considered significant only if there was a fold change ≥ 1.5 or ≤ 0.67. Heatmap graphs were performed using R software (version 3.6.3, R Foundation for Statistical Computing, Vienna, Austria). The protein abundance was transformed into the logarithmic format (log_2_Abundance) and normalized. Euclidean distance was used for hierarchical clustering analysis.

## Supplementary information

Supplementary Information.

Supplementary Data S1.

Supplementary Data S2.

Supplementary Data S3.
