# Supplementary material for: Investigation of structural proteins in sea cucumber (Apostichopus japonicus) body wall
Source: Sci Rep. 2020 Oct 30;10:18744. doi: 10.1038/s41598-020-75580-x (PMC7599334; doi:10.1038/s41598-020-75580-x)
Supplement: Supplementary file 1 — Supplementary Information. [file 41598_2020_75580_MOESM1_ESM.pdf]

Supplementary material

Investigation of structural proteins in sea cucumber (*Apostichopus japonicus*) body wall

Yanchao Wang[a], Mo Tian[a], Yaoguang Chang[a,b*], Changhu Xue[a,b], Zhaojie Li[a]

[a] College of Food Science and Engineering, Ocean University of China, 5 Yushan Road, Qingdao, 266003, China

[b] Laboratory for Marine Drugs and Bioproducts, Qingdao National Laboratory for Marine Science and Technology, Qingdao, 266237, China

[*] Corresponding author. E-mail address: changyg@ouc.edu.cn; Tel.: + 86-532-82032597

Data S1: List of *in silico* predicted structural proteins (ECM proteins, muscle proteins and proteases) of sea cucumber (*Apostichopus japonicus*).

Data S2: List of identified structural proteins (ECM proteins, muscle proteins and proteases) in the sea cucumber body wall by using an iTRAQ-based proteomics approach. Tables shaded with gray color represented proteins containing only one peptide and those proteins were excluded from protein quantification data.

Data S3: List of structural proteins with significantly different abundances (fold change ≥ 1.5 or ≤ 0.67) among samples from different spatial locations of sea cucumber body wall.

Figure S1: Sample collection from the sectioned sea cucumber body wall.

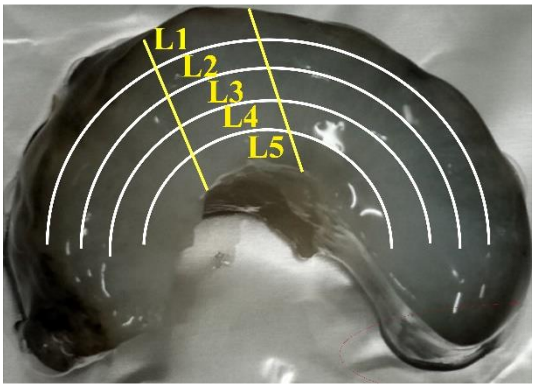

Figure S1
